# Severe haemolytic anaemia and acute renal failure caused by pinhole perforation of native mitral valve: a case report

**DOI:** 10.1093/ehjcr/ytaf290

**Published:** 2025-06-27

**Authors:** Ayami Naito, Yuji Nagatomo, Satonori Maekawara, Risako Yasuda, Koji Tsutsumi, Fumihiko Kimura, Hiroo Kumagai, Takeshi Adachi

**Affiliations:** Department of Cardiology, National Defense Medical College, 3-2 Namiki, Tokorozawa, Saitama 359-8513, Japan; Department of Cardiology, National Defense Medical College, 3-2 Namiki, Tokorozawa, Saitama 359-8513, Japan; Department of Cardiology, National Defense Medical College, 3-2 Namiki, Tokorozawa, Saitama 359-8513, Japan; Department of Intensive Care, National Defense Medical College, 3-2 Namiki, Tokorozawa, Saitama 359-8513, Japan; Department of Cardiovascular Surgery, National Defense Medical College, 3-2 Namiki, Tokorozawa, Saitama 359-8513, Japan; Department of Hematology, National Defense Medical College, 3-2 Namiki, Tokorozawa, Saitama 359-8513, Japan; Department of Nephrology, National Defense Medical College, 3-2 Namiki, Tokorozawa, Saitama 359-8513, Japan; Department of Cardiology, National Defense Medical College, 3-2 Namiki, Tokorozawa, Saitama 359-8513, Japan

**Keywords:** Case report, Haemolytic anaemia, Renal failure, Mitral valve perforation, Acute heart failure, Cardiac magnetic resonance

## Abstract

**Background:**

Mechanical haemolytic anaemia following mitral valve plasty or replacement is not uncommon. However, to our knowledge, there are no reports of haemolytic anaemia caused by native mitral valve regurgitation requiring surgical intervention.

**Case summary:**

A 70-year-old woman was admitted for acute decompensated heart failure with moderate mitral regurgitation and haemolytic anaemia. Although her heart failure responded promptly to medical therapy, her renal function progressively deteriorated, ultimately requiring haptoglobin supplementation. Haematologic conditions potentially causing haemolysis were excluded, and mitral regurgitation (MR) was suspected as the underlying cause. Cardiac magnetic resonance imaging and transoesophageal echocardiography identified an MR jet through a pinhole perforation of the A3 segment of the mitral valve, which was subsequently confirmed intraoperatively. The patient’s haemolytic anaemia improved markedly following mitral valve replacement. We concluded that the mechanical haemolysis was due to MR through a pinhole perforation of the native mitral valve.

**Discussion:**

A prior study suggested the presence of subclinical intravascular haemolysis in patients with primary MR. In the present case, an accelerated MR jet through a pinhole perforation, in addition to a jet directed against the atrial wall, appears to have caused clinically significant haemolysis. This case highlights that native mitral valve perforation can induce mechanical haemolysis in a manner similar to that seen following mitral valve surgery.

Learning points

- Native valve perforation can cause mechanical haemolysis similarly to post-mitral valve surgery. Multimodality imaging can help identify occult abnormalities leading to the development of mechanical haemolytic anaemia.
- Valvular heart disease with native valve should be considered as a cause of haemolytic anaemia

## Primary specialties involved other than cardiology

Cardiovascular surgery, haematology, nephrology.

## Introduction

Mechanical haemolysis is a recognized complication following heart valve repair, although it rarely necessitates re-operation. Acute renal failure secondary to haemolysis is uncommon but can have serious consequences when it occurs. To our knowledge, mechanical haemolytic anaemia resulting from native mitral valve regurgitation (MR) has not previously been reported. Herein, we present a rare case of a woman admitted with acute decompensated heart failure who was found to have haemolytic anaemia due to native mitral valve regurgitation and severe acute renal failure.

## Summary figure

**Figure:**
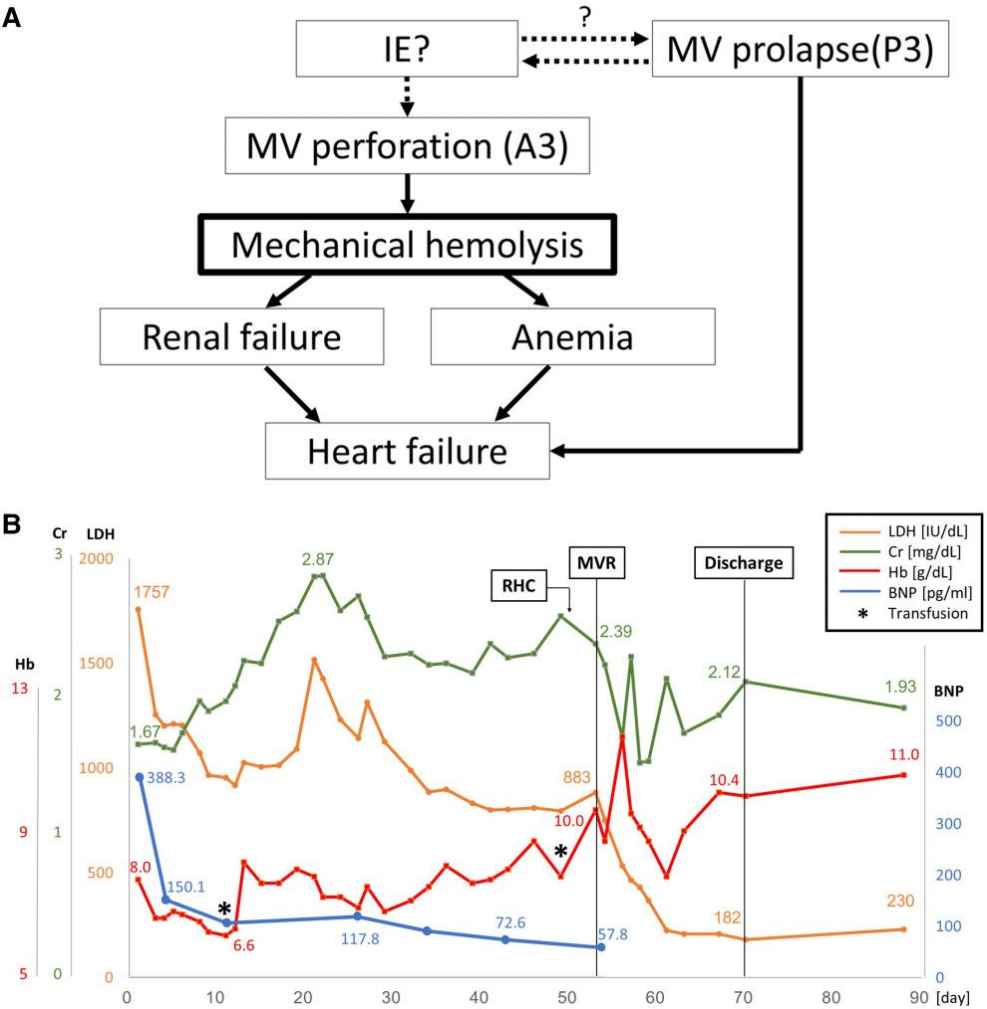
(*A*) The proposed mechanisms leading to haemolysis which resulted in heart failure. (*B*) Temporal changes of laboratory values. Despite the resolution of heart failure, renal function progressively worsened day by day from the day of admission. After surgery, LDH markedly decreased and Hb level improved. LDH, lactate dehydrogenase; Cr, creatinine; Hb, haemoglobin level; BNP, B-type natriuretic peptide.

## Case presentation

A 70-year-old woman was referred to our hospital with acute decompensated heart failure, macrocytic anaemia, and acute kidney injury. She reported fatigue and loss of appetite persisting for 2 months prior to admission, which she had attributed to her pre-existing depression. She had been under long-term follow-up by her family doctor for ∼20 years following a kidney donation; laboratory data 18 months prior to admission showed near-normal estimated glomerular filtration rate (eGFR) and haemoglobin (Hb) levels.

**Figure 1:**
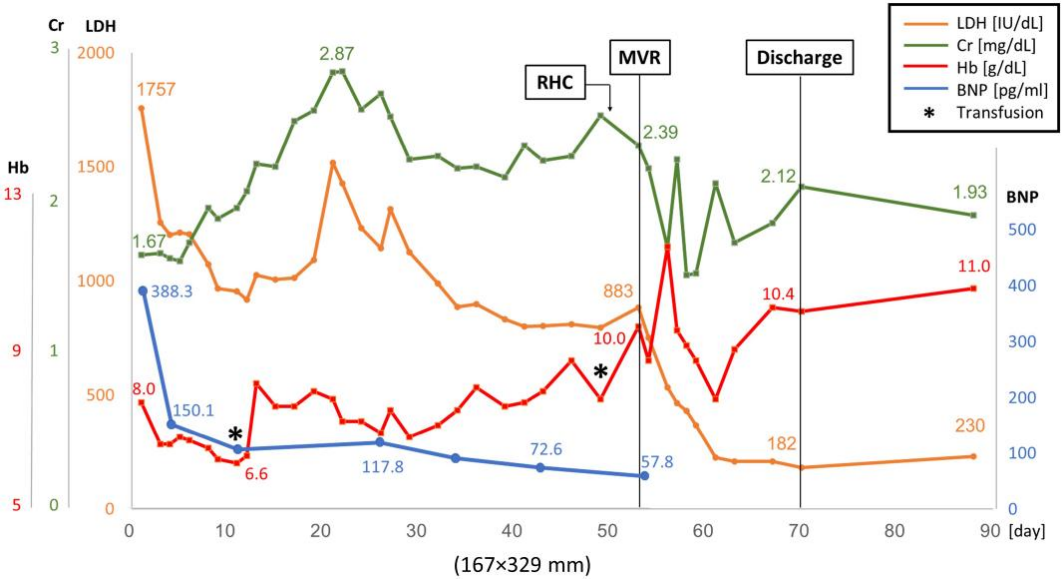
Temporal changes of laboratory values. Despite the resolution of heart failure, renal function progressively worsened day by day from the day of admission. After surgery, LDH markedly decreased and Hb level improved. LDH, lactate dehydrogenase; Cr, creatinine; Hb, haemoglobin level; BNP, B-type natriuretic peptide.

**Figure 2:**
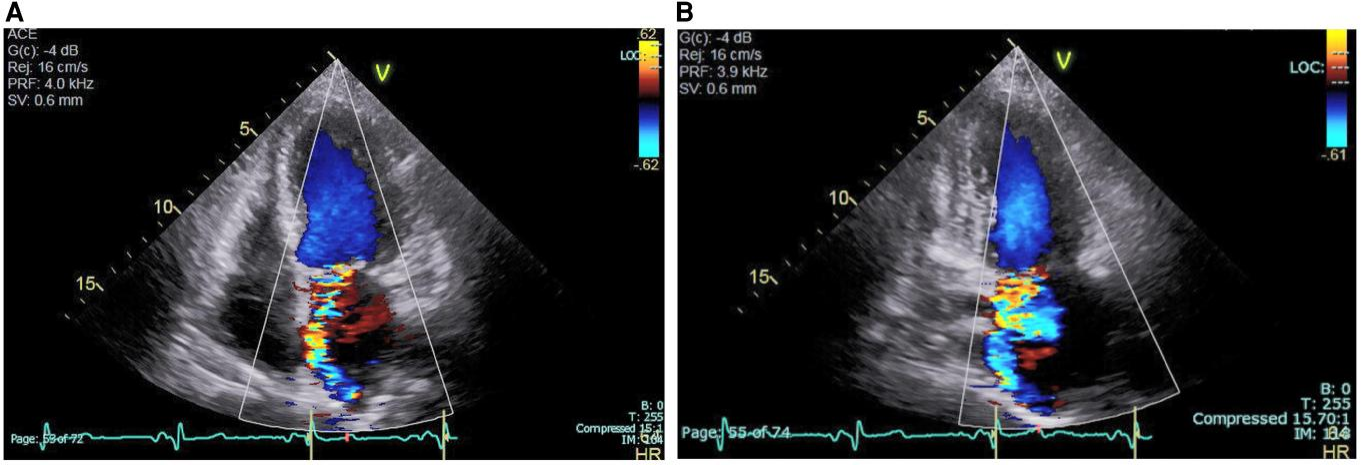
Transthoracic echocardiography. Colour Doppler imaging showed eccentric mitral regurgitation in (*A*) apical four-chamber and (*B*) two-chamber view.

**Figure 3:**
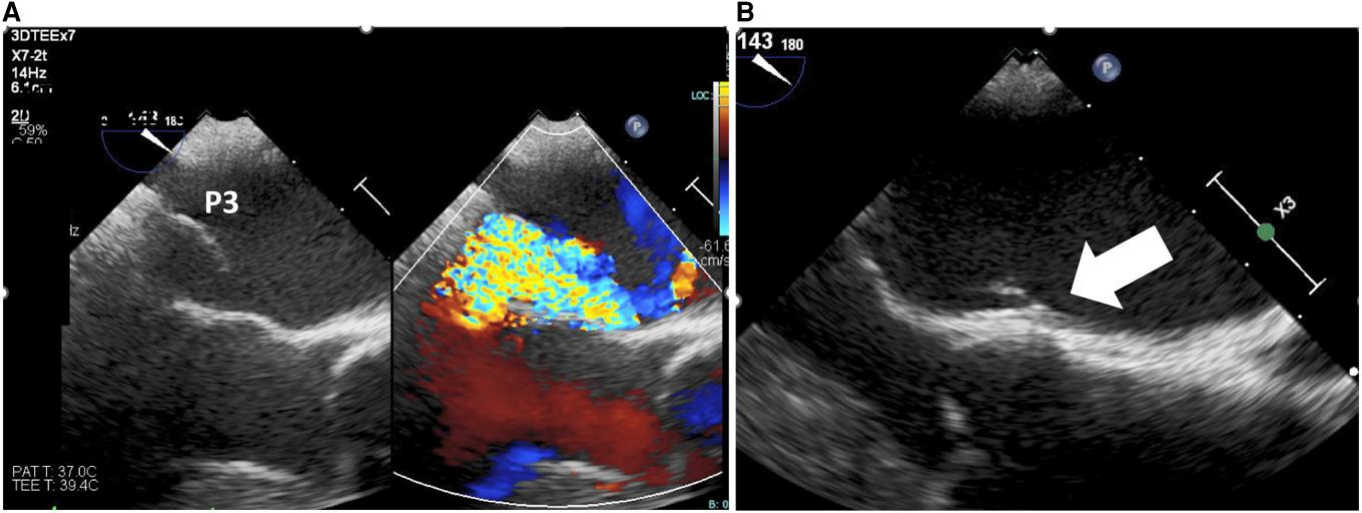
First transoesophageal echocardiography. (*A*) Mid-oesophageal aortic valve long-axis view showed mitral regurgitation due to P3 prolapse. (*B*) Magnified image of the same view showed vegetation on atrial septum where the mitral regurgitation jet impinged (arrow).

**Figure 4:**
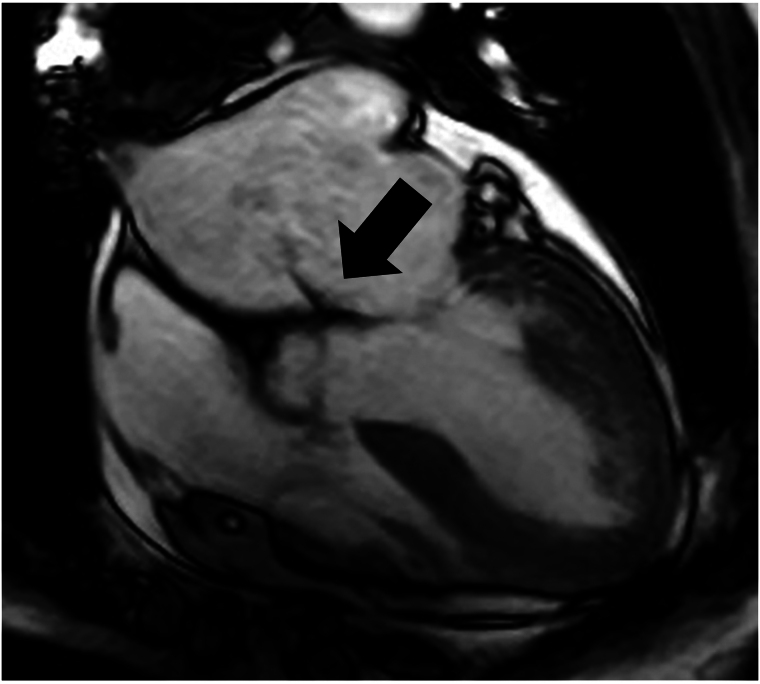
Cardiac magnetic resonance imaging (CMR). CMR cine showed a fast narrow jet through anterior mitral leaflet (arrow).

**Figure 5:**
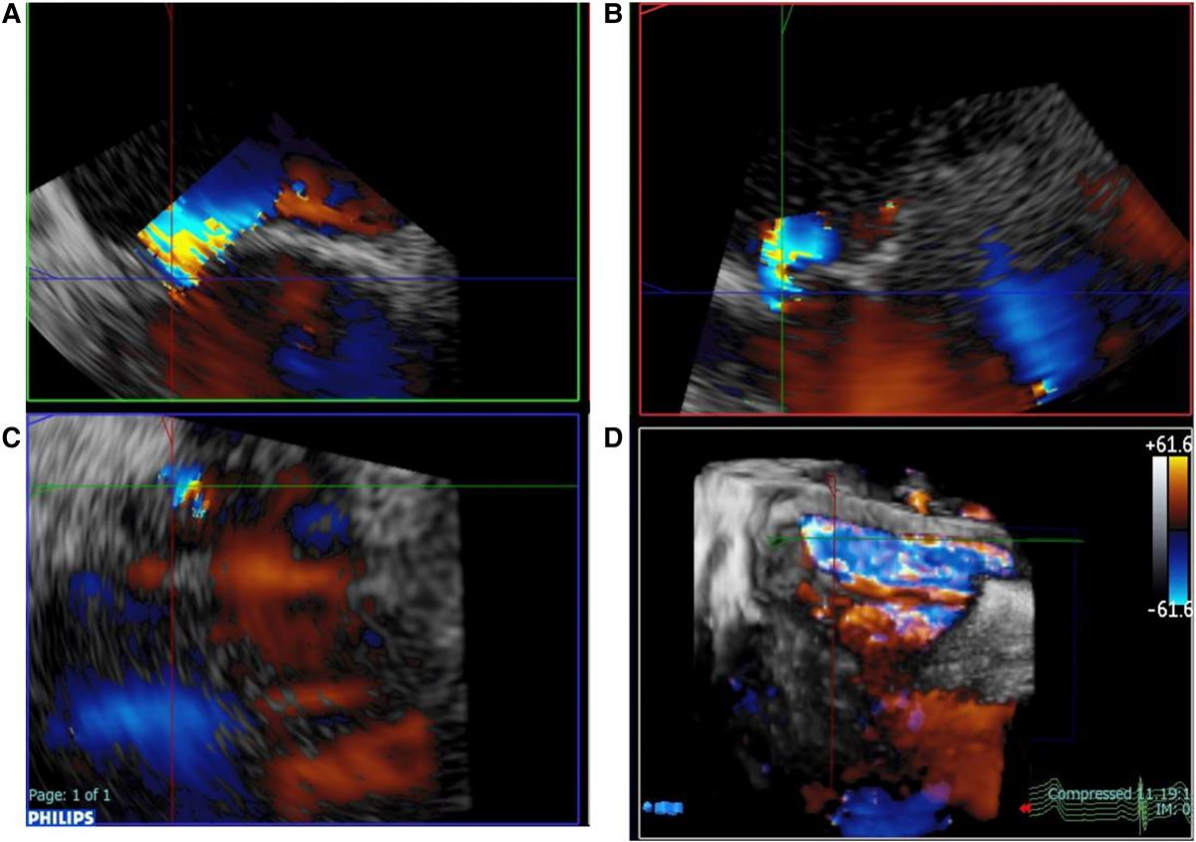
Second transoesophageal echocardiography. Commissure (*A*), long-axis (*B*), and short-axis (*C*) views reconstructed from three-dimensional image (*D*). Reconstructed images showed narrow mitral regurgitation jet through a pinhole perforation at the A3 segment.

On admission, the patient presented with leg oedema and mild dyspnoea. Initial laboratory tests showed elevated B-type natriuretic peptide (BNP) at 388.3 pg/mL (normal range: <18.5 pg/mL) (*Figure 1*). Further findings included red blood cell count 2.11 × 10^7^/μL (normal 3.80–5.10 × 10^7^/μL), haemoglobin 8.0 g/dL (normal 12.0–16.5 g/dL), haematocrit 24.1% (normal 35%–45%), mean corpuscular volume 114.2 fL (normal 85–100 fL), reticulocyte count 10‰ (normal 5–20‰), undetectable haptoglobin, mildly elevated ferritin with normal serum iron and total iron-binding capacity, total bilirubin 2.8 mg/mL (normal 0.3–1.2 mg/dL) (conjugated/unconjugated 0.87/1.93), lactate dehydrogenase (LDH) 1757 IU/L (normal 119–229 U/L), and C-reactive protein 0.4 mg/dL (normal < 0.3 mg/dL). A peripheral blood smear showed fragmented erythrocytes. The creatinine level was 1.67 mg/mL (eGFR 24.2 mL/min/1.73 m^2^, normal > 60 mL/min/1.73 m^2^), and urinary haemoglobin and haemosiderin were detected (*Figure 1* and *Summary figure B*). There was no evidence of inflammation or infection. The patient’s vitamin B12 level was within normal limits (425 mg/mL, normal 233–914 pg/mL), and folate was below the normal range (1.4 ng/mL, normal 3.6–12.9 ng/mL). Although folate deficiency was initially suspected as the cause of anaemia, haemolysis persisted despite folate supplementation. Differential diagnoses included autoimmune haemolytic anaemia, paroxysmal nocturnal haemoglobinuria, thrombotic microangiopathy, and other haematologic conditions including haematopoietic malignancies. However, investigations—including Coombs test, expression of CD55 and CD59 on erythrocytes, ADAMTS13 activity, and bone marrow aspiration—revealed no abnormalities. Multiple blood cultures were negative. Mechanical haemolysis was subsequently suspected. Transthoracic echocardiography (TTE) showed preserved left ventricular ejection fraction and MR with suspected posterior leaflet (P3) prolapse (*Figure 2*). Quantification suggested severe MR (regurgitant volume 100 mL, effective regurgitant orifice area 0.7 cm^2^). Transoesophageal echocardiography (TEE) revealed moderate to severe eccentric MR due to P3 prolapse (vena contracta 6.8 mm, pulmonary vein reverse flow not documented) with vegetation on the atrial septum on which the MR jet impinged (*Figure 3*). The severity of MR may have been underestimated due to the haemodynamic changes induced by sedation during TEE. Healed infective endocarditis (IE) was suspected based on these findings, although MR from the P3 prolapse alone was considered an unlikely cause of haemolysis at that stage. Cardiac magnetic resonance (CMR) was performed to identify possible abscesses or other IE-related abnormalities. Unexpectedly, CMR revealed a narrow, high-velocity MR jet through the mitral leaflet (*Figure 4*, Supplementary material online, *Video S1*). A second TEE with very careful observation confirmed a narrow MR jet through a pinhole perforation at the A3 segment of the anterior mitral leaflet (*Figure 5*, Supplementary material online, *Video S2*).

**Figure 6:**
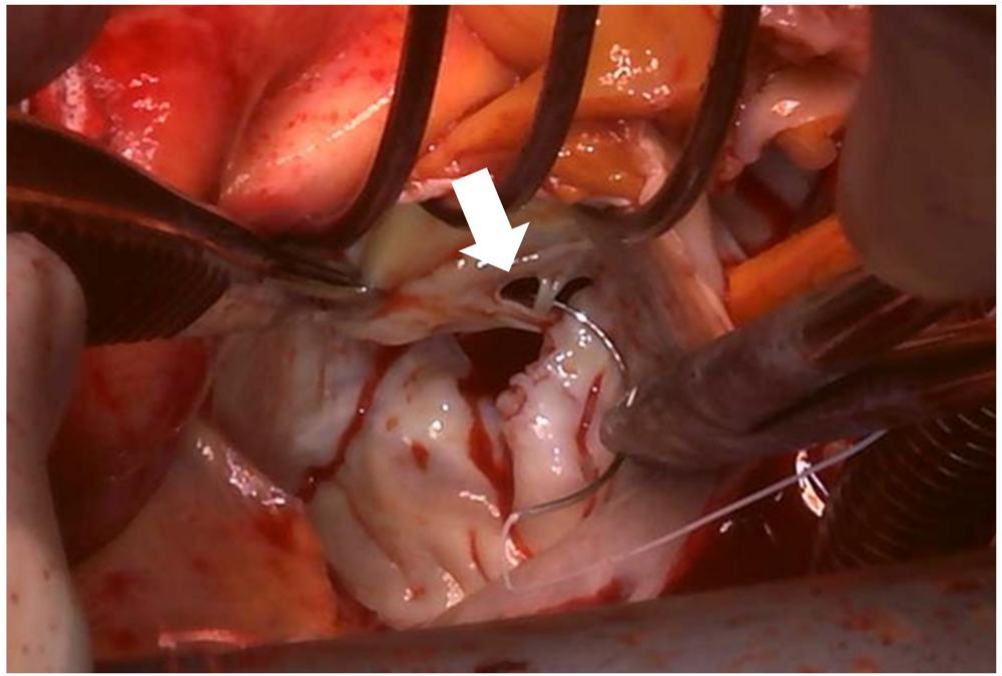
Intraoperative photograph. A small perforation was detected on A3 segment (arrow).

**Figure 7:**
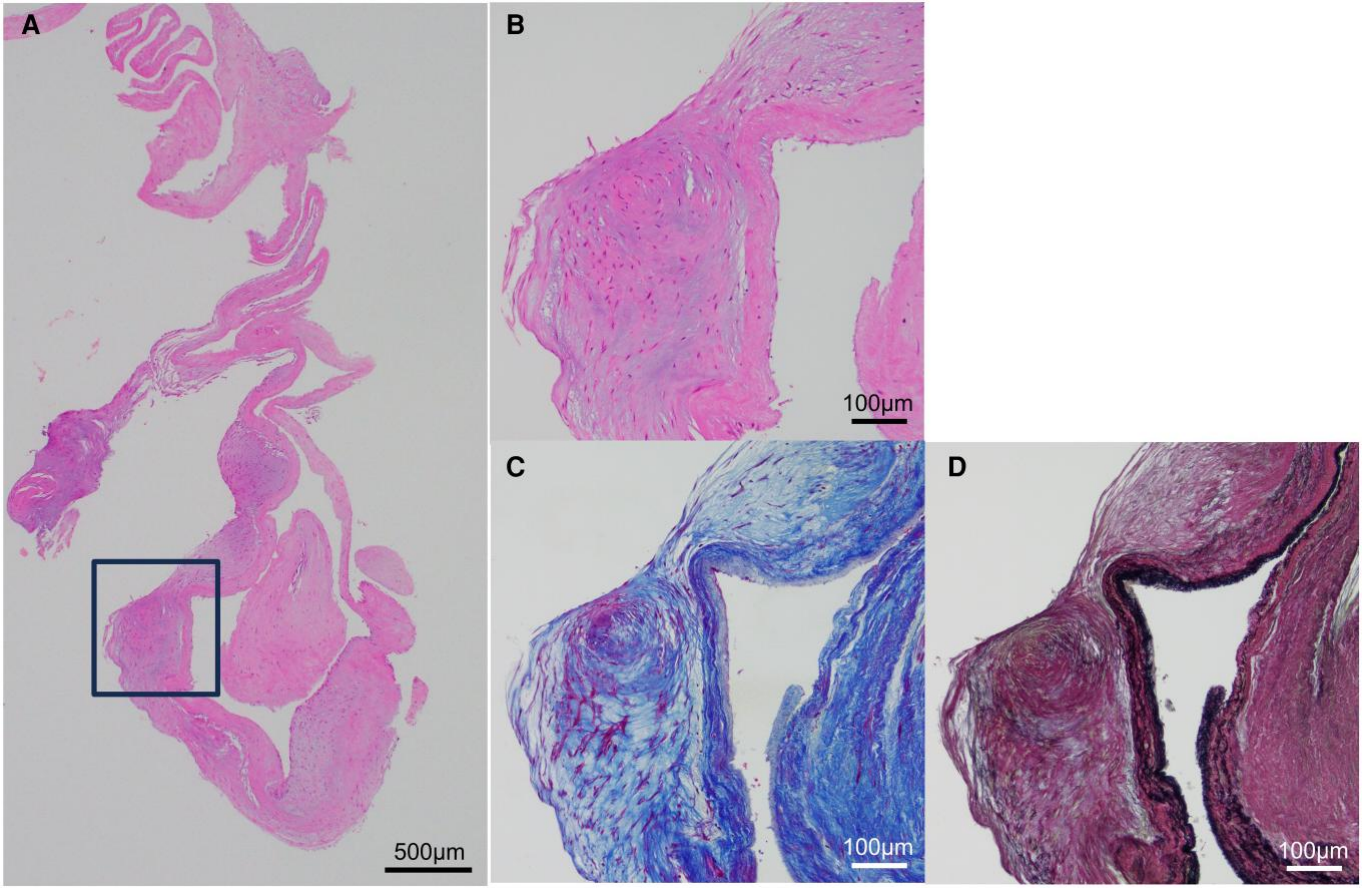
The pathology of vegetation on atrial septum. (*A*) Haematoxylin eosin staining in the low power field. (*B*) Haematoxylin eosin staining, (*C*) Masson Trichrome staining, and (*D*) Elastica Van Gieson staining in the high-power field. The pathology showed fibroblasts hyperplasia and thickened fibrous tissue with areas of vitrification, which was consistent with healed IE. IE, infective endocarditis.

Following admission, the patient’s heart failure was treated using intravenous furosemide, which resulted in prompt clinical improvement and reduction in BNP (*Figure 1* and *Summary* f*igure B*). Right heart catheterization on Day 50 revealed stable haemodynamics, although pulmonary artery wedge pressure showed a pronounced *v* wave (see Supplementary material online, *Figure S1*). Despite resolution of heart failure, renal function continued to decline (*Figure 1* and *Summary figure B*), requiring haptoglobin supplementation. Based on our strong suspicion that haemolysis was caused by the MR jet through the pinhole perforation, mitral valve replacement (MVR) was undertaken. Intraoperative inspection confirmed a pinhole perforation at A3 (*Figure 6*), and the valve was successfully replaced with a 27-mm St. Jude Medical prosthesis. After the operation, the patient’s haemolytic anaemia rapidly improved (*Figure 1* and *Summary figure B*; LDH, creatinine, haemoglobin level). Histopathology of the excised valve showed no additional perforations, and the atrial septal vegetation was consistent with healed IE, showing fibroblast proliferation and thickened fibrous tissue with areas of vitrification (*Figure 7*). The postoperative course was uneventful. The patient was discharged on postoperative Day 14 and has remained stable, with no recurrence of heart failure or haemolysis. Her serum creatinine level stabilized at 2.0 to 2.5 mg/dL (eGFR 14–19 mL/min) during outpatient follow-up.

## Discussion

We report a rare case of haemolytic anaemia and renal failure that improved significantly following MVR. These findings suggest that mechanical haemolysis due to an MR jet through a pinhole perforation in the native mitral valve contributed to the clinical presentation.

Mechanical haemolysis is a recognized complication after mitral valve surgery,^1,2^ although severe cases requiring re-operation are rare.^3^ Proposed mechanisms include fragmentation of a jet by an intervening solid structure, collision of a jet against a solid structure, and acceleration of blood flow through a small orifice (<2 mm),^4–7^ all of which can induce erythrocyte destruction and subsequent phagocytosis by macrophages in the reticuloendothelial system.^8^

To our knowledge, there have been no case reports of severe mechanical haemolytic anaemia caused by native valve MR and requiring surgery. In native valves, haemolysis is considered rare in the absence of prosthetic material, although subclinical intravascular haemolysis has been described in primary MR.^9^ While acquired von Willebrand syndrome is another recognized cause of intravascular haemolysis in native valve pathology (e.g. with aortic stenosis),^10^ ADAMST13 activity was normal in our patient. We propose that haemolysis in the present case was primarily caused by mechanical stress from the accelerated MR jet through the pinhole perforation, although MR from the P3 prolapse may have contributed. IE was suspected as the underlying cause of the perforation (*Summary figure A*). The pre-existing P3 prolapse may have contributed to the development of IE by creating a turbulent jet that impinged on the atrial septum, as visualized on TEE (*Figure 2B*). Conversely, IE itself may have led to the P3 prolapse via chordal rupture (*Summary figure A*), although this was not supported by imaging or histologic findings. The patient had no typical symptoms of IE, such as fever before admission; however, a small proportion of patients can reportedly develop IE without fever.^11^ Inflammatory markers were low at presentation (C-reactive protein 0.4 mg/dL). The timing of IE onset remains unclear, and investigation for IE-induced peripheral embolization was limited due to progressive renal dysfunction.

Cardiac magnetic resonance provides superior spatial resolution for cardiac assessment, comparable to TEE in evaluating valvular regurgitation in both native^12^ and prosthetic valves. In the present case, CMR enabled identification of the MR jet through the pinhole perforation, which had been overlooked in TTE and initial TEE. Advancements in time and spatial resolution of CMR may increase its utility in the assessment of valvular heart disease.

Current guidelines recommend re-operation in cases of prosthetic valve regurgitation causing haemolysis requiring repeated transfusions or associated with severe heart failure symptoms (class I),^13,14^ with catheter-based percutaneous repair as an alternative for high-risk patients (class IIa).^13,14^ In our case, we suspected that haemolytic anaemia triggered heart failure, although direct evidence was lacking. Surgical indication based solely on MR severity was unclear, as the patient’s heart failure stabilized with medical therapy. Ultimately, the decision for surgery was based on two factors: (i) MR through the pinhole perforation was the only plausible cause of the haemolysis and decompensated heart failure, and (ii) renal function continued to deteriorate despite all interventions. Although MV plasty is typically preferred in cases of primary MR, including leaflet perforations, we opted for MVR due to concerns about identifying and addressing all perforations intraoperatively.

## Conclusion

We report an exceptionally rare case of severe haemolytic anaemia caused by native MV regurgitation through a small perforation, leading to acute renal failure. This case highlights that native valves may also cause mechanical haemolysis, even in patients without a history of cardiac surgery. Valvular heart disease should be considered a potential cause in patients presenting with unexplained haemolytic anaemia.

## Supplementary Material

ytaf290_Supplementary_Data

## Data Availability

Data underlying this article will be shared upon reasonable request to authors.

## Lead author biography

Dr Ayami Naito graduated from National Defense Medical College in 2016. After her residency, she has been involved in clinical cardiology practice. She has a special interest in echocardiographic research of heart failure. Alongside her clinical practice, she trained as a military physician, receiving education in primary care and military medicine. In 2024, she started basic research on vascular disease as a postgraduate student.
