# Supplementary figures and images for: Severe haemolytic anaemia and acute renal failure caused by pinhole perforation of native mitral valve: a case report
Source: Eur Heart J Case Rep. 2025 Jun 27;9(7):ytaf290. doi: 10.1093/ehjcr/ytaf290 (PMC12215661; doi:10.1093/ehjcr/ytaf290)

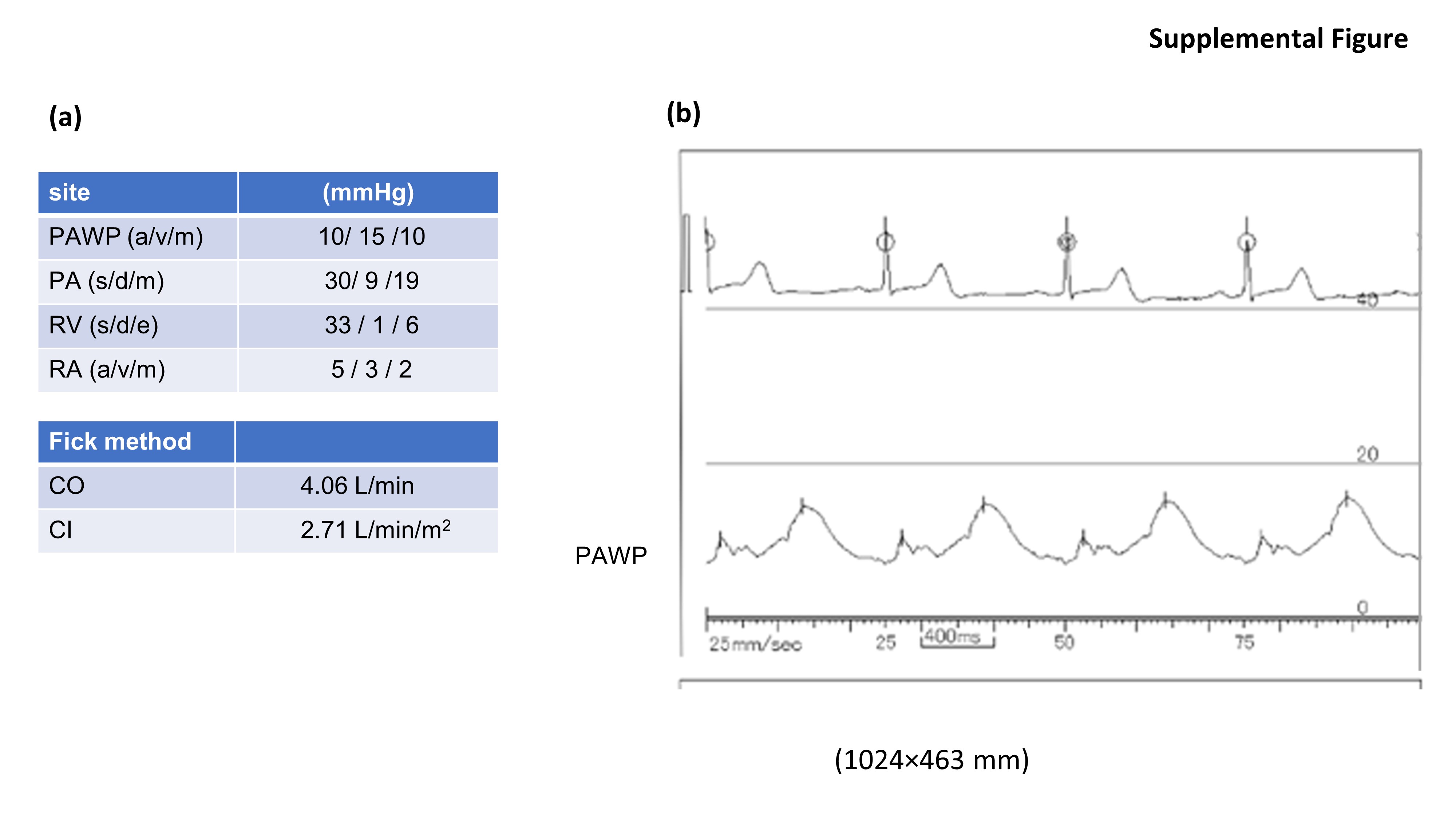

Supplement: ytaf290_Supplementary_Data [file ytaf290_Supplementary_Data.zip › Supplemental Figure.JPG]
